# Factors associated with underreporting of adverse drug reactions by patients: a systematic review

**DOI:** 10.1007/s11096-023-01592-y

**Published:** 2023-05-29

**Authors:** Catarina Costa, Patricia Abeijon, Daniela A. Rodrigues, Adolfo Figueiras, Maria Teresa Herdeiro, Carla Torre

**Affiliations:** 1 Faculdade de Farmácia da Universidade de Lisboa, Lisbon, Portugal; 2 Facultad de Medicina y Odontología de la Universidad de Santiago de Compostela, A Coruña, Galicia Spain; 3 Research Unit for Inland Development, Polytechnic Institute of Guarda (UDI-IPG), Guarda, Portugal; 4 Health Sciences Research Centre, University of Beira Interior (CICS-UBI), Covilhã, Portugal; 5 Department of Preventive Medicine and Public Health, University of Santiago de Compostela, Santiago de Compostela, Spain; 6 Health Research Institute of Santiago de Compostela (IDIS), University of Santiago de Compostela, Santiago de Compostela, Spain; 7 Consortium for Biomedical Research in Epidemiology and Public Health (CIBER Epidemiology and Public Health - CIBERESP), Madrid, Spain; 8 Department of Medical Sciences, Institute of Biomedicine - iBiMED, University of Aveiro, Aveiro, Portugal; 9 Laboratory of Systems Integration Pharmacology, Clinical and Regulatory Science, Research Institute for Medicines (iMED.ULisboa), Lisbon, Portugal

**Keywords:** Adverse drug reactions, Pharmacovigilance, Spontaneous reporting, Underreporting

## Abstract

**Background:**

Spontaneous reporting is the most used method to monitor post-marketing safety information. Although patient involvement in spontaneous reporting has increased overtime, little is known about factors associated with patients’ adverse drug reaction (ADR) reporting.

**Aim:**

To identify and assess the sociodemographic characteristics, attitudes and knowledge that influence spontaneous reporting and the reasons associated with ADR underreporting by patients.

**Method:**

A systematic review was conducted according to PRISMA guidelines. A search on the MEDLINE and EMBASE scientific databases was performed to retrieve studies published between 1 January 2006 and 1 November 2022. Studies were included if they addressed knowledge and attitudes associated with ADR underreporting.

**Results:**

A total of 2512 citations were identified, of which 13 studies were included. Sociodemographic characteristics were frequently identified with ADR reporting in 6 studies, being age (3/13) and level of education (3/13) the most often reported. Older age groups (2/13) and individuals with higher level of education (3/13) were more likely to report ADRs. Underreporting was shown to be motivated by reasons related to knowledge, attitudes, and excuses. Ignorance (10/13), complacency (6/13), and lethargy (6/13) were the most frequent reasons for not reporting.

**Conclusion:**

This study highlighted the scarcity of research conducted with the aim of assessing ADR underreporting by patients. Knowledge, attitudes, and excuses were commonly observed in the decision to report ADRs. These motives are characteristics that can be changed; hence strategies must be designed to raise awareness, continually educate, and empower this population to change the paradigm of underreporting.

**Supplementary Information:**

The online version contains supplementary material available at 10.1007/s11096-023-01592-y.

## Impact statements

- Approaches to increasing ADR reporting should consider the sociodemographic characteristics of patients, including the level of education.
- Strategies to decrease ignorance, complacency, and lethargy seem to be necessary to address the lack of reporting culture of ADR by patients.
- The continuous implementation of awareness campaigns and educational programs tailored to different knowledge levels, could increase the engagement, and encourage the active participation of patients.
- More research is required to inform the public, enhance ADR reporting and to provide current evidence on the effectiveness of pharmacovigilance interventions in reporting practice.

## Introduction

Adverse drug reactions (ADRs) are a significant and worldwide public health problem, being a frequent cause of increased mortality [1–5], morbidity [1–4], healthcare costs [5–9] and hospital admissions [3, 5–9]. Spontaneous reporting is the most widely used method in pharmacovigilance to monitor safety information after a drug has been marketed, and through the collection of administrative health data provides the largest volume of information [1, 10, 11].

Although in the early years of pharmacovigilance, reporting ADRs was restricted to healthcare professionals (HCPs), HCPs and patients can now report suspected ADRs to spontaneous reporting systems [12, 13]. In 2012, there was a major change at the European Commission level, with the publication of pharmacovigilance legislation: Directive 2010/84/EU and Regulation No 1235/2010 [14]. One of the major changes comprised the empowerment and emphasis given to citizens as an additional source of information, enabling them to directly report suspected ADRs [15–17].

It is acknowledged that the type of information reported by patients and HCPs is different [18]. Patients’ reports provide new information, including more subjective and detailed symptom descriptions of how ADRs impact daily life. HCPs report more objective and clinically related information [19]. The information provided by both populations demonstrates a positive contribution to obtain a more complete and full knowledge of reported ADRs [15, 20–22].

ADRs are significantly underreported worldwide, with estimates that more than 94% are not reported by HCPs [23]. Underreporting can delay the detection of safety signals, making it difficult to evaluate and quantify risk factors, and consequently, compromising the full knowledge of the drug safety profile [11, 23, 24]. Furthermore, although direct patient reporting has been increasing over time, the overall contribution is still very low, representing only 9% of the total reports in 2014 [19, 25–28].

Little is known about factors associated with ADR reporting by patients; however, studies have been emerging in recent years. Identifying the main barriers is crucial to understand gaps and to design specific strategies that can positively impact the quantity and quality of ADR reports and ultimately increase the safety of medicines. One systematic review was performed in 2006 regarding patient reporting of suspected ADRs, however none of the included studies concerned spontaneous reporting [29]. More recently, a systematic review assessed the motives and/or barriers that influence patient reporting of ADRs [30]. Our review assessed the factors associated with underreporting of ADR, including sociodemographic characteristics, reasons, and attitudes (modelled using Inman’s seven deadly sins [31–33]), as well methodological quality, thereby adding more information to the body of evidence. Together with previous reviews, this covers the entire period of direct patient reporting worldwide.

### Aim

The purpose of this systematic review was to identify and assess the sociodemographic characteristics, attitudes and knowledge that influence spontaneous reporting, and the reasons associated with underreporting of ADRs by patients.

## Method

This systematic review was performed according to the Preferred Reporting Items for Systematic Reviews and Meta-Analyses (PRISMA) 2020 guidelines [34] and a research protocol was registered in PROSPERO network database (CRD42021227944).

### Search strategy

A search of MEDLINE PubMed and EMBASE scientific databases was performed to retrieve articles published between 1 January 2006 and 1 November 2022, with final search conducted on 2 November 2022. The search strategy used was: (attitud* OR knowledge* OR barrier* OR facilitators*) AND (Adverse Drug Reaction Reporting Systems[MesH] OR Drug-Related Side Effects and Adverse Reactions[MeSH]) AND report* (Electronic supplement material 1). The references cited by the included studies were examined by manual search.

### Eligibility criteria

Studies were considered eligible for inclusion if: (i) the target population were patients and/or consumers; (ii) written in English, French, Portuguese, or Spanish; (iii) aimed to assess factors (personal characteristics as well as the reasons) associated with ADR underreporting; (iv) addressed ADR reporting through spontaneous reporting. The studies included could be either observational or interventional as they comprised original data. However, in interventional studies only baseline data were collected.

Conference abstracts/proceedings, reviews, editorials, letters to the editor, comments, theses/dissertations, systematic reviews and/or meta-analysis were excluded. Studies in which the target population were HCPs and/or students, focused on a specific pathology or treatment, and performed through intensive monitoring schemes or clinical trials were also excluded. For studies with no access, the authors were contacted. Finally, studies that identified attitudes and knowledge but did not directly associate underreporting reasons were excluded.

All articles retrieved were independently screened by 2 reviewers (CC and PA or CC and DR), who conducted full-text analysis to assess suitability for inclusion. For any divergent decisions, a third reviewer (CT, TH or AF) acted as referee to reach consensus.

### Risk of bias and quality assessment

Critical assessment of the quality and risk of bias of the included studies were assessed using the Appraisal tool for Cross-Sectional Studies (AXIS) [35]. This tool was created in 2016 to appraise reporting quality, study design quality and risk of bias. It consists of a 20-component questionnaire, in which 7 questions focus on assessing the quality of the reports, 7 on the study design, and the remaining 6 assess the possibility of bias in the study. Fifteen out of the twenty items cover the methods and results sections, which reveals the importance of these two sections for critical appraisal of this tool. Two reviewers (CC and PA or CC and DR) independently conducted the critical assessment for each study, and in case of disagreement, a third reviewer (CT, TH or AF) was responsible for resolving discrepancies.

### Data extraction and synthesis

Data from the eligible studies were extracted based on Lopez-Gonzalez et al. [36]. Reasons for ADR underreporting were based on a list of 7 attitudes described as the “seven deadly sins” (i.e., complacency, diffidence, ignorance, indifference, lethargy, greed: financial incentives, fear and guilty). This list was proposed in 1976 and further amended in 1986 and in 1996 [31–33]. Additionally, to update and complement this list, other reasons influencing ADR underreporting were added according to those described in the literature. Therefore, 4 categories were created: (i) attitudes relating to professional activities (e.g., financial incentives, legal aspects); (ii) problems associated with ADR-related knowledge and attitudes (e.g., fear, complacency, diffidence, indifference, ignorance, insecurity); (iii) excuses made by the individual (e.g., lethargy); and (iv) others (e.g., lack of feedback, ADR resolved, poor reporting system). All these categories are described in the Electronic supplement material 2.

The extraction was performed by 2 authors (CC and PA or CC and DR) independently and included: author (year of publication), country, study design, setting, study population, response rate, sample size, data source, personal characteristics associated to reporting and reasons for not reporting ADRs. In case of inconsistencies, a third reviewer (CT, TH or AF) made the final decision. If further information was needed the corresponding authors were contacted.

### Data analysis

The characteristics of the included studies were assessed through a descriptive analysis with each reason of underreporting reported numerically as a percentage (i.e*.* either extracted directly from the study (one specific question for each reason) or calculated using the mean/median when more than one question within the same study was associated to the same reason category mentioned above).

## Results

### Study selection

**Fig. 1:**
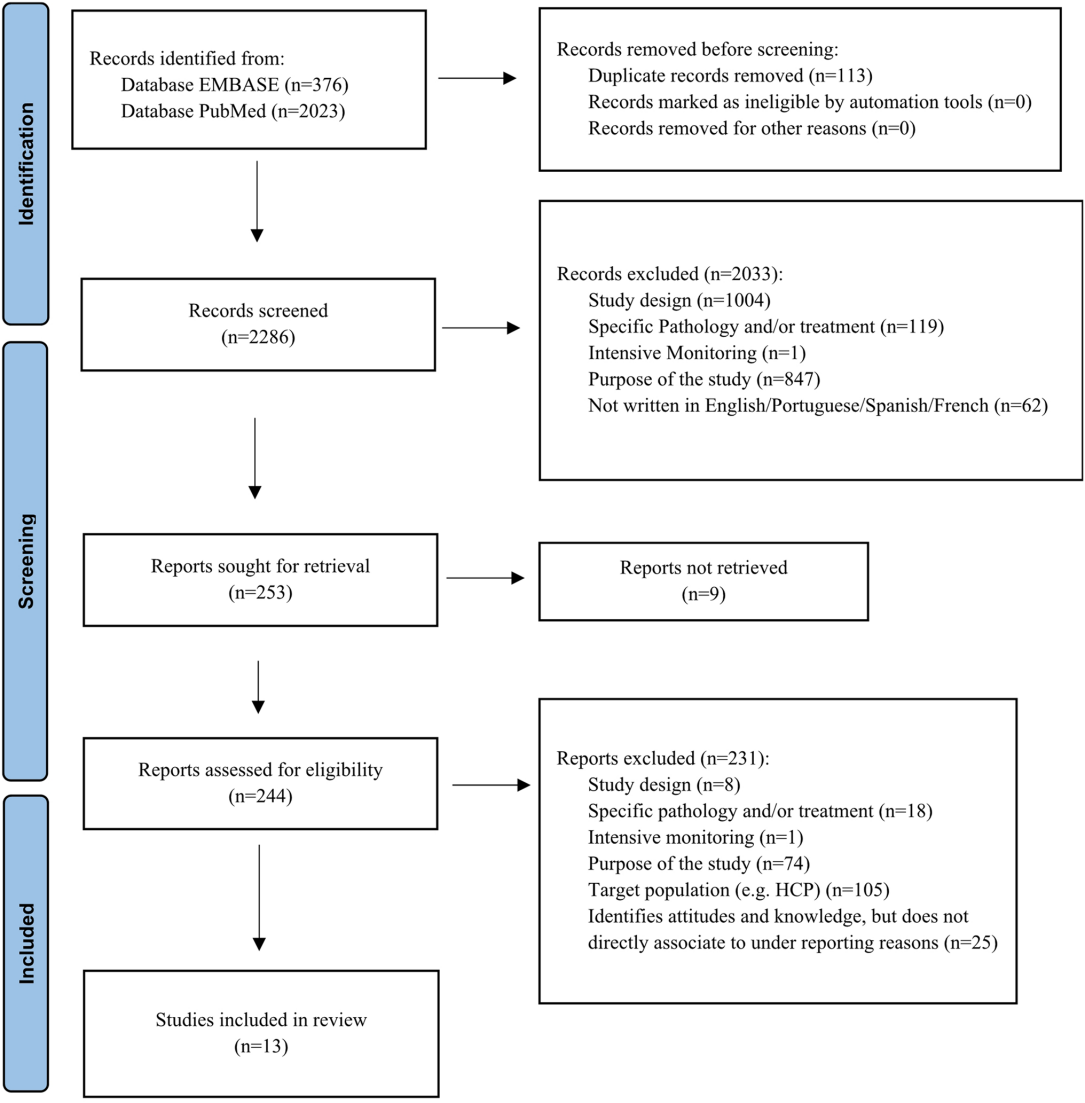
PRISMA 2020 flow diagram summarizing the study selection process

A total of 2399 citations were obtained from MEDLINE PubMed (n = 2023) and EMBASE (n = 376). After screening titles and abstracts, 113 duplicate studies were removed, and 2286 citations potentially met the inclusion criteria. Since 9 studies were excluded due to inaccessibility of full-text, a total of 253 articles underwent to full-text analysis. This resulted in thirteen studies [37–49] meeting the inclusion criteria and were included in the review (Fig. 1).

### Characteristics of selected studies

The general characteristics of included studies are presented in Table 1 and the main study findings in Table 2.

**Table 1:** Characteristics of the included studies that assessed factors associated with adverse drug reactions underreporting by patients

Author (Year of publication)	Country	Study design^b^	Setting	Sample size	Response rate (%)	Data collection method	Scale	Quality assessment (score obtained/total score)^c^
Wang et al*.* (2022) [37]	China	Cross sectional	Not mentioned	869	80.09%	Directly administered	Likert	17/20
Januskiene et al. (2021)^a^ [38]	Several countries	Not mentioned	Not mentioned	1138	Not applicable	Internet	Multiple choice; free text	14/20
Pillay et al*.* (2021) [39]	South Africa	Quantitative descriptive	Not mentioned	206	Not applicable	Internet	Multiple choice; free text	18/20
Al Dweik et al. (2020) [40]	Canada	Qualitative	Not mentioned	15	Not applicable	Interview	Free text	16/20
Kim et al*.* (2020) [41]	South Korea	Cross sectional	Not mentioned	1000	100%	Internet	Likert	17/20
Adisa and Omitogun (2019)^a^ [42]	Nigeria	Cross sectional	Primary care	360	94.7%	Interview	Free text; multiple choice	19/20
Adisa et al*.* (2019) [43]	Nigeria	Cross sectional	Hospital	1190	99.1%	Interview; directly administered	Free text; multiple choice	18/20
Jacobs et al*.* (2019) [44]	Ghana	Quantitative and qualitative	Hospital; primary care; retail	572	100%	Interview; Directly administered	Not mentioned	17/20
Sabblah et al*.* (2017) [45]	Ghana	Cross sectional	Retail	510	86.7%	Interview	Likert	15/20
Patsuree et al*.* (2016) [46]	Thailand	Cross sectional	Hospital; primary care; retail	2400	81.8%	Directly administered	Likert, free text, visual analogue scale	15/20
Matos et al*.* (2015) [47]	Portugal	Descriptive-correlational study	Retail	1337	81.1%	Directly administered	Likert	15/20
Robertson and Newby (2013) [48]	Australia	Cross sectional	Not mentioned	4981	6%	Internet	Not mentioned	10/20
Fortnum et al*.* (2012) [49]	United Kingdom	Not mentioned	Not mentioned	2028	6.5%	Interview	Not mentioned	9/20

^a^Article with information on HCPs and patients, however only patient information was retrieved

^b^Study designs as reported by the authors

^c^Quality of the studies was assessed using the Appraisal tool for Cross-Sectional Studies (AXIS)

**Table 2:** Studies that assessed the sociodemographic characteristics and reasons associated with underreporting adverse drug reactions

Author (Year of publication)	Sociodemographic characteristics^b^	Reasons for under-reporting adverse drug reactions^c^			
		Complacency	Ignorance	Lethargy	Others
Wang et al*.* (2022) [37]		NR	72.58%	16.49%	10.71%
Januskiene et al. (2021)^a^ [38]		NR	23.99%	3.69%	✔
Pillay et al*.* (2021) [39]		NR	37.38%	17.00%	✔
Al Dweik et al. (2020) [40]		✔	✔	NR	✔
Kim et al*.* (2020) [41]	Sex, monthly income	NR	71.30%	NR	63.50%
Adisa and Omitogun (2019)^a^ [42]	Age	14.70%	27.40%	NR	NR
Adisa et al*.* (2019) [43]		NR	NR	NR	15.40%
Jacobs et al*.* (2019) [44]	Age, region	NR	✔	NR	✔
Sabblah et al*.* (2017) [45]	Age, sex, level of education, profession/employment status	14.80%	10.80%		37.00%
Patsuree et al. (2016) [46]		NR	70.40%	17.10%	42.90%
Matos et al. (2015) [47]	Level of education	24.60%	NR	NR	NR
Robertson and Newby (2013) [48]		35.60%	NR	16.40%	26.40%
Fortnum et al*.* (2012) [49]	Social grade, level of education	22.00%	16.20%	7.40%	14.80%

✔—In studies where the reasons were stated but whose numerical measures were not mentioned, the symbol ✔ was used

*NR* Not reported

^a^Article with information on HCPs and patients, however only patient information was retrieved

^b^Sociodemographic characteristics reported to be statistically significantly associated with ADR reporting

^c^The total numerical percentage regarding each reason for underreporting ADRs within each study was either extracted directly from the study (one specific question for each reason) or calculated using the mean/median (i.e. when more than one question within the same study was associated to a certain reason)

Five studies were conducted in Africa [39, 42–44, 50], 3 in Europe [38, 47, 49], 3 in Asia [37, 41, 46], 1 in Australia [48] and 1 in Canada [40]. Most of the studies (n = 8, 61.5%) were cross-sectional, with 2 [38, 47, 49] failing to report the study design.

Four studies [44–47] were conducted in a retail/community pharmacy, followed by 3 studies [43, 44, 46] in hospital and 3 [42, 44, 46] in primary care.

The sample size ranged from 15 to 4981 subjects and the response rate from 6.0 to 100%, being above 50.0% in 8 of the studies. The study with the highest number of recruited subjects (n = 4981) had the lowest response rate (6.0%) [48].

Six studies were conducted through direct interviews, which were face-to-face [42–45] or telephone calls [49] or both [40], 5 studies opted to directly administer the questionnaire on site [37, 43, 44, 46, 47] and 4 studies used a web-based survey [38, 39, 41, 48]. Two studies combined 2 different types of data collection methods [43, 44].

Likert scale was the most often used (n = 5) [37, 41, 45–47]. The free text response was used in 6 studies [38–40, 42, 43, 46], followed by multiple choice (n = 4) [38, 39, 42, 43], visual analog scale (VAS) (n = 1) [46], with 3 studies failing to report the type of scales used [44, 48, 49].

### Quality assessment

Six studies [37, 39, 41–44] met ≥ 17 AXIS criteria, and therefore were considered of high methodological quality and low susceptibility to bias. Five studies [38, 40, 45–47] were evaluated as having a moderate level of methodological quality and risk of bias, and the remaining of low methodologically quality and high susceptibility to bias. In general, the criteria least met were sample size justification, selection process and measures to address non-responders. None of the studies included any description of non-responders (Electronic supplement material 3).

### Sociodemographic characteristics

The two most prevalent factors associated with the act of reporting were the level of education (n = 3) and age (n = 3). Age did not reach consensus since 1 study [42] revealed that the younger population was more likely to report ADRs than older age groups, whereas 2 studies [44, 50] showed the opposite. In all studies, individuals with higher levels of education were more likely to report ADRs.

The remainder reported factors were sex [41, 45], region [44], monthly income [41], social grade [49] and profession/employment status [50]. Specifically, it was found that males [41, 45], subjects living in urban areas (*vs* rural areas) [44], with higher monthly income [41] and higher social grades [49] were associated with higher intention to report ADRs. Additionally, a significant association was found between private sector employees and self-employed subjects and the higher likelihood to report as compared to unemployed, student, government employee and retired people [50].

### Reasons for underreporting ADRs

The reasons that influenced the reporting of ADRs by patients were restricted to four categories. Ignorance (n = 10; with values ranging between 10.8 and 72.6%) [37–42, 44–46, 49] about the identification/recognition of ADRs, the possibility and importance of reporting them, as well as not knowing the needed requirements to report. Complacency (n = 6; values ranging from 14.7 to 35.6%) [40, 42, 45, 47–49] mostly related with the conviction that only safe drugs are on the market and that serious ADRs are well documented by the time the drug is marketed. Lethargy (n = 6; values ranging from 3.7 to 17.1%) [37–48, 48, 49, 49–81], with patients referring that ADR reporting is time-consuming, generates work and that they would do it if the process was easier. In this category, forgetfulness and procrastination were also mentioned, but at a lower frequency. Other reasons (n = 11) [37–41, 43–46, 48, 49] that made patients reluctant to report ADRs included: (i) responsibility for ‘reporting lies’ with HCPs, (ii) ADR resolved or already expected; (iii) stopped taking the medicine; (iv) absence or difficulty in communicating with the HCP (e.g*.* unsupportive physician, diminishing ADR importance, lack of guidance, feeling it has to be reported to a physician and not another HCP); (v) lack of feedback or action taken by the authorities regarding previously submitted reports; and (vi) being abroad. Two studies did not present descriptive statistics for their assessment [40, 44].

## Discussion

### Main findings

This review showed that sociodemographic characteristics were commonly observed with ADR reporting whereas knowledge, attitudes, and excuses were frequently identified with ADR underreporting by patients. This review also highlighted the scarcity of research conducted with the aim of assessing ADR underreporting by patients. Over the last 15 years, most of the studies were conducted in African countries, where the pharmacovigilance framework is weak and relatively new [51]. The individual case safety reports retrieved by African countries represents only < 1% of the global reports [52] and to overcome this, patient reporting initiatives have been developed [53]. Over the review period, only 3 studies were conducted in European countries, despite the permission to allow ADR reporting by patients since 2012. Australia was the first country to allow direct patient reporting (1964) [12, 13] and had last evaluated this topic in 2013. Although Canada, USA and New Zealand shortly followed Australia [12], only Canada recently conducted one study to assess reasons for ADR underreporting.

### Strengths and weaknesses

Our review has some limitations. Firstly, a search for studies in grey literature was not carried out. However, we believe that our results would not be affected since studies with this type of objective are usually associated with academic institutions where the incentive and ambition for publication is higher. We decided not to include conference abstracts/proceedings and editorials as these materials often reflect preliminary analysis, and it is less likely that methods and results are described with the necessary detail for the purpose of our study. Moreover, the percentages associated with each barrier might be subject to bias as means/medians were calculated if two or more statements were grouped in the same category within the same study. Finally, we acknowledge that our systematic review is limited by the details that authors have reported. However, an assessment of quality was performed for all included studies. Despite these acknowledged limitations, we believe that our main findings are relevant, and the data collected are comprehensive to assess the factors associated with underreporting of ADRs, thereby adding information to the body of evidence.

### Interpretation of study findings

Regarding sociodemographic characteristics, the age factor was not unanimous. One study showed that the younger population is more likely to report ADRs [42], as they are well informed and may be of their relatives who might be more susceptible to experience an ADR. Since this population is a consumer of technologies, they can have a great impact on both learning and information sharing [13, 54]. However, 2 studies [44, 45] reported quite the opposite, as expected given that age is a well-known predisposing factor for the occurrence of ADRs [55]. Concerning education levels, all 3 studies [45, 47, 49] revealed that individuals with higher levels of education were more likely to report ADRs, a finding which is in line with the literature [56, 57].

Reasons related to ignorance were widely reported as obstacles to ADR reporting. These reasons may be associated with the fact that they have never heard about pharmacovigilance and may not recognize its importance. This ignorance may also be described as poor knowledge dissemination given that reporting systems might be more aligned with HCPs than patients [58].

Complacency was another reported reason as patients might believe that an ADR is not serious enough, or that it is not necessary to report. Complacency and ignorance could be highly related to the belief that marketed drugs are well-documented and safe. Lethargy justifies statements about forgetfulness, procrastination, lack of time or interest to report, and difficulty filling out the report. Simplifying reporting, promoting easier access, and boosting strategies that demonstrate that it is neither burdensome nor a bureaucratic process can overcome this barrier.

Overall, previous studies conducted among HCPs showed a wider spectrum of reasons for underreporting: fear, indifference, diffidence, ignorance, complacency, insecurity, unavailability of the reporting form, lethargy, ambition, and financial reimbursement [36, 42, 59–65]. The discrepancy in the number of reasons given by HCPs and patients for underreporting may be associated with their different levels of knowledge concerning pharmacovigilance.

As in our review, patient-HCP communication problems have been identified in other studies [20, 66–68] and might explain the reported barriers given by HCPs [62, 69–79] concerning the patients' lack of information to submit reports or even disapproval by HCPs to patient reporting [80]. This aspect could be improved by creating a patient-friendly environment, giving an openness to speak without embarrassment or fear of judgment, thus positively changing the patient-HCP relationship.

A previous systematic review identified additional reasons for not reporting such as postal mailing costs or the limitation of restricting reporting to telephone during working hours [30]. However, these barriers probably no longer apply given the alternative methods of reporting ADRs (e.g*.* on-line platforms, mobile apps), which do not have associated costs or time limitations [13]. Of note, countries with an online reporting system option were associated with higher patient reporting rates [28].

The lack of interventions for patients was highlighted in a systematic review [81] which comprised 28 studies conducted with the aim of assessing if interventions targeting HCPs and patients were effective in improving ADR reporting. Only 1 study targeted patients, highlighting the need to develop tailored interventions for this population.

Several altruistic and personal motives that encourage patients to report ADRs have been described, such as contributing to increase safety knowledge, the need to be heard and to prevent harm to other patients [45, 47, 57, 66]. Other motives reported were related to the severity of the ADR, asking for feedback, feeling uncomfortable sharing information with HCPs [36, 59, 60] or the fact that HCPs did not take the case seriously [43, 44, 47, 57].

From the methodological point of view, only 6 studies of our systematic review were considered of high quality and low susceptibility to bias, which emphasizes the need for improvement when conducting future studies. Most of the included studies used a convenience sample and representativeness may be lacking, leading to potential selection bias. Most studies did not present a denominator and although some studies attempted to address non-responder rates, none was completely successful.

### Future research

There is an urgent need to develop and conduct high quality, intervention based studies in this population to optimize ADR reporting and to provide evidence of effectiveness. Different communication, educational and promotional strategies targeted to patients should be designed and implemented to overcome the potentially modifiable barriers. Training and dissemination related to online platforms with clear, simple, and interactive content could be an efficient option to overcome these barriers.

## Conclusion

This review highlighted the scarcity of studies conducted with the aim of assessing the influencing factors of underreporting ADRs by patients.

Sociodemographic characteristics appear to influence spontaneous reporting, with age and level of education being the most frequently reported. Patients’ attitudes observed linked to underreporting included ignorance, complacency, lethargy. These can be improved through the implementation of awareness campaigns and theoretical/practical educational programs tailored to different knowledge levels. Increasing engagement and encouraging active participation of patients is needed to reverse the current paradigm of underreporting.

### Supplementary Information

Below is the link to the electronic supplementary material.

Supplementary file1 (DOCX 32 KB)
