# Supplementary material for: Factors associated with underreporting of adverse drug reactions by patients: a systematic review
Source: Int J Clin Pharm. 2023 May 29;45(6):1349–58. doi: 10.1007/s11096-023-01592-y (PMC10682061; doi:10.1007/s11096-023-01592-y)
Supplement: Supplementary file 1 — Supplementary file1 (DOCX 32 KB) [file 11096_2023_1592_MOESM1_ESM.docx]

**Supplementary Information (SI)**

**Electronic supplement material 1**: EMBASE and PubMed full search strategy

| **EMBASE ALL<Database baseline to 1 November 2022>**  **Search date: 2 November 2022** | | |
| --- | --- | --- |
| **#** | **Searches** | **Results** |
| 1 | (attitud* or knowledge* or barrier* or facilitators*).af. | 2 051 775 |
| 2 | report*.af. | 7 674 794 |
| 3 | (adverse and reactions).af. | 127 534 |
| 4 | ('drug related' and side and effects).af. | 5 918 |
| 5 | 'drug-related side effects'.af. | 2 480 |
| 6 | 'adverse reactions'.af. | 53 536 |
| 7 | 3 or 6 | 127 534 |
| 8 | 4 or 5 | 5 918 |
| 9 | 7 and 8 | 1 935 |
| 10 | ('drug' exp or drug).af. | 12 395 743 |
| 11 | ('reaction' exp or reaction).af. | 4 052 532 |
| 12 | ('reporting' exp or reporting).af. | 355 855 |
| 13 | adverse.af. | 2 389 566 |
| 14 | systems.af. | 1 568 608 |
| 15 | 10 and 11 and 12 and 13 and 14 | 2 046 |
| 16 | 'adverse drug reaction reporting systems'.af. | 293 |
| 17 | 9 or 15 or 16 | 3 945 |
| 18 | 1 and 2 and 17 | 428 |
| 19 | remove published before 1 January 2006 from 18 | 376 |
| **PubMed ALL<1 January 2006 to 1 November 2022>**  **Search date: 2 November 2022** | | |
| **#** | **Searches** | **Results** |
| 1 | (attitud* OR knowledge* OR barrier* OR facilitators*) AND (Adverse Drug Reaction Reporting Systems[MesH] OR Drug-Related Side Effects and Adverse Reactions[MeSH]) AND report* | 2 023 |

**Electronic supplement material 2:** Reasons for ADR underreporting based on Inman´s “seven deadly sins” and identified in literature (e.g., other systematic reviews, included studies)

|  | **REASONS** | **EXPLANATION** |
| --- | --- | --- |
| **Professional**  **activities** | Ambition^1^ | To publish personal case series |
|  | Financial reimbursement^1^ | Belief that there should be a financial incentives or reward for reporting |
|  | Legal aspects^1^ | Fear of possible involvement in a lawsuit |
| **Knowledge**  **and**  **attitudes** | Fear^1^ | - Fear of damaging relationships and negative impact on the company that produced or marketed the drug - Fear of confidentiality issues |
|  | Complacency^1^ | Conviction that only safe drugs are on the market and that serious ADRs are well documented when the drug is marketed |
|  | Ignorance^1^ | - Lack of knowledge to recognise the ADR and its importance - Lack of knowledge about the requirements needed to report, where to report, how to describe the notification and how the information in further used; belief that only serious or unexpected ADRs should be reported |
|  | Indifference^1^ | Belief that one case could not contribute to medical knowledge |
|  | Diffidence^1^ | - Lack of confidence and fear of appearing ridiculous for making a report of ADRs merely based on a suspicion - Only reports if it is sure that it was drug-related |
|  | Insecurity^2^ | The belief that it is nearly impossible to have certain that a particular drug was responsible for causing a particular adverse reaction |
|  | Unavailability of the reporting form^2^ | Do not have access to the report form |
| **Excuses** | Lethargy^1^ | - Procrastination and postponing the notification - Lack of time or motivation, effort, or interest to report - Need for an easier method - The report will generate extra work - Forgetfulness |
| Others**^2^** | | - Lack of feedback on the report submitted - Stopped taking the medicine - ADR resolved - Lack of clinical training - Lack of communication - Not their responsibility for reporting - Lack of confidence in the regulatory authority - Poor reporting system |

*^1^Proposed by Inman*

*^2^Describe in the literature*

**Electronic supplement material 3:** Critical assessment of quality and risk of bias of included studies using the AXIS tool

| Author  (Year of publication) | AXIS criterion | | | | | | | | | | | | | | | | | | | | |
| --- | --- | --- | --- | --- | --- | --- | --- | --- | --- | --- | --- | --- | --- | --- | --- | --- | --- | --- | --- | --- | --- |
|  | **1** | **2** | **3** | **4** | **5** | **6** | **7** | **8** | **9** | **10** | **11** | **12** | **13** | **14** | **15** | **16** | **17** | **18** | **19** | **20** |  |
| Wang et al. (2022) |  |  |  |  |  |  | **X** |  |  |  |  |  | **X** | **X** |  |  |  |  |  |  |  |
| Januskiene et al. (2021) |  |  | **X** |  |  |  | **X** |  |  | **X** | **X** |  | **X** | **X** |  |  |  |  |  |  |  |
| Pillay et al. (2021) |  |  |  |  |  |  | **X** |  |  |  |  |  |  | **X** |  |  |  |  |  |  |  |
| Dweik et al. (2020) |  |  |  |  |  |  | **X** |  |  | **X** |  |  | **X** | **X** |  |  |  |  |  |  |  |
| Kim et al. (2020) |  |  |  |  |  | **X** |  |  |  |  |  | **X** |  | **X** |  |  |  |  |  |  |  |
| Adisa and Omitogun (2019) |  |  |  |  |  |  |  |  |  |  |  |  |  | **X** |  |  |  |  |  |  |  |
| Adisa et al. (2019) |  |  |  |  |  | **X** |  |  |  |  |  |  |  | **X** |  |  |  |  |  |  |  |
| Jacobs et al. (2019) |  |  |  |  |  |  | **X** |  |  |  |  |  | **X** | **X** |  |  |  |  |  |  |  |
| Sabblah et al. (2017) |  |  | **X** |  | **X** | **X** | **X** |  |  |  |  |  |  | **X** |  |  |  |  |  |  |  |
| Patsuree et al. (2016) |  |  |  |  | **X** | **X** | **X** |  |  |  | **X** |  |  | **X** |  |  |  |  |  |  |  |
| Matos et al. (2015) |  |  | **X** |  | **X** | **X** | **X** |  |  |  |  |  |  | **X** |  |  |  |  |  |  |  |
| Robertson and Newby (2013) |  |  | **X** |  |  | **X** | **X** | **X** | **X** | **X** | **X** |  | **X** | **X** | **X** |  |  |  |  |  |  |
| Fortnum et al. (2012) |  | **X** | **X** |  |  | **X** | **X** |  | **X** |  | **X** |  |  | **X** |  |  | **X** | **X** | **X** | **X** |  |

***Blank cell:*** *The study complied with the question.*

***Cell marked with X:*** *The study did not comply with the question.*
